# Association between serum omentin-1 concentrations and body composition measured by dual-energy X-ray absorptiometry in Japanese elementary school-aged children

**DOI:** 10.1186/s40101-025-00406-6

**Published:** 2025-11-07

**Authors:** Yuki Murakami, Yuki Fujita, Kumiko Ohara, Harunobu Nakamura, Masayuki Iki, Katsuyasu Kouda

**Affiliations:** 1 Present Address: Department of Hygiene and Public Health, Faculty of Medicine, Kansai Medical University, 2-5-1 Shin-Machi, Hirakata, Osaka 573-1010 Japan; 2 Department of Epidemiology for Community Health and Medicine, Kyoto Prefectural University of Medicine, 465 Kajii-cho, Kawaramachi-Hirokoji, Kamigyo, Kyoto 602-8566 Japan; 3 Department of Public Health, Faculty of Medicine, Kindai University, 377-2 Oono-Higashi, Osaka-Sayama, Osaka, 589-8511 Japan

**Keywords:** Adipokines, Fat mass parameters, Children, Dual-energy X-ray absorptiometry, Epidemiology

## Abstract

**Background:**

Omentin-1 (also known as intelectin-1) is a novel adipokine associated with metabolic diseases. However, its physiological role in body composition remains incompletely understood. Therefore, this study aimed to investigate the association between the circulating omentin-1 levels and whole-body and regional body composition parameters measured using dual-energy X-ray absorptiometry (DXA).

**Methods:**

A population-based cross-sectional survey was conducted among school-aged children in Hamamatsu, Japan. Serum adipokine levels were measured using enzyme-linked immunosorbent assay, and associations between omentin-1 levels and DXA-based parameters were evaluated by multiple regression analysis after adjusting for potential confounding factors.

**Results:**

The final study included 392 participants (192 boys, 200 girls, 75.2% of the source population; mean age 11.2 ± 0.3 years). Serum omentin-1 levels showed a significantly inverse association with nearly all DXA-based fat mass parameters. Inverse correlations were observed with fat-free soft tissue mass and serum leptin levels, whereas positive correlations were noted with adiponectin levels. The mean values for various body fat parameters, fat-free soft tissue mass, body mass index, and waist circumference were significantly decreased across tertiles of serum omentin-1 levels from lowest to the highest after adjusting for potential confounders.

**Conclusion:**

Our results demonstrate that Japanese school-aged children with higher fat mass tended to have lower serum omentin-1 levels. These findings provide crucial insights into the link between omentin-1 levels and body composition, which may contribute to early health interventions for metabolic improvement.

## Background

Adipokines, such as leptin, adiponectin, and omentin-1 (also known as intelection-1), are bioactive proteins produced by adipose tissue. The expression and secretion of these adipokines vary by fat depot, particularly between visceral adipose tissue (VAT) and subcutaneous adipose tissue (SAT). Numerous studies have explored the effects of depot-specific differences on health. In general understanding, VAT surrounds the organs in the abdominal cavity and has been linked to an increased risk of metabolic disorders and cardiovascular diseases [1].

Omentin-1, a novel adipokine, was initially identified as a VAT depot-specific secretory protein through a human omental fat cDNA library [2]. Early experimental studies also demonstrated that omentin-1 mRNA expression is predominantly localized to VAT, with minimal expression in SAT. Animal studies have further shown that VAT from high-fat diet-fed animals exhibits reduced omenti-1 levels compared with those in the control diet group [3, 4]. However, conflicting evidence exists. For instance, omentin-1 gene deletion in mice did not increase susceptibility to diet-induced obesity, suggesting a minor role for omentin-1 in this process [5]. Additionally, studies involving type 2 diabetes mellitus (T2DM) mouse models reported no changes in plasma omentin-1 levels [6]. In contrast, human studies indicated that circulating omentin-1 levels decrease in obese condition and increase following weight loss [7, 8]. Lower omentin-1 levels have been observed in individuals with impaired glucose regulation and untreated T2DM compared with those with normal glucose tolerance [9]. These findings underscore a disparity between animal and human data, necessitating further research to clarify the physiological and pathophysiological roles of omentin-1.

While overweight status and obesity are characterized by excessive fat accumulation, most previous studies that have investigated omentin-1 adopted body mass index (BMI) as a surrogate for adiposity rather than direct fat mass measurements to define overweight and obesity as an outcome variable [3, 9, 10]. However, BMI has limitations, as it does not differentiate between fat and fat-free masses. Alternative methods such as air displacement plethysmography, bioelectrical impedance analysis (BIA), hydrostatic weighing, and dual-energy X-ray absorptiometry (DXA) offer direct assessment of body composition. Among them, DXA stands out for its precision, safety, and applicability in both children and adults. A recent systematic review identified DXA to be more valid than BIA for estimating body composition, further establishing DXA as the preferred method for measuring fat mass in clinical and research settings [11].

To the best of our knowledge, no population-based study has investigated the relationship between serum omentin-1 levels and body composition measured by DXA. Therefore, this epidemiological study aimed to investigate the association between serum omentin-1 levels and DXA-measured body composition in school-aged children. By examining these associations, the study seeks to advance understanding of the physiological role of omentin-1.

## Methods

### Study populations

A cross-sectional survey was conducted between November and December of 2010 and 2011 in Hamamatsu City, Japan. The study population included all fifth-grade students (n = 521; 268 boys and 253 girls) enrolled in Aritama Elementary School and Sekishi Elementary School. As no private schools existed in the district, these schools represented nearly all school-aged children residing in the study area. Comprehensive data were collected for each participant, including blood samples, total and regional fat mass measurements, current maternal BMI, sedentary behavior, and pubertal status (assessed by the appearance of pubic hair).

Parents and guardians received printed information outlining the study procedures, including the dose of radiation exposure associated with DXA scans. DXA was chosen due to its high precision and specific advantages for pediatric assessment. Written consent was obtained from all parents or guardians prior to enrollment this study, and students were allowed to decline participation voluntarily. The study was approved by the Ethics Committee of Kansai Medical University (Approval No. 2021250) and conducted in accordance with the ethical principles of the Declaration of Helsinki.

### Anthropometry and body composition measurements

Body weight and height were measured while participants wore light clothing, without shoes. Hairstyles and accessories were removed to ensure accurate height measurements. Height was recorded with participants standing upright; with heels together; back, buttocks, and heels touching the stadiometer’s post; arms relaxed at the sides; and head positioned with the eyes and ears aligned horizontally [12]. Waist circumference was measured following the protocol recommended by the Ministry of Health, Labor, and Welfare [13].

Whole-body and regional-body composition were assessed using a mobile DXA scanner (QDR-4500A; Hologic Inc., Bedford, MA) in a bus transported to each school. The DXA scans followed previously established protocols [13]. Participants wore light clothing, free of metal objects, during the examination. A single experienced medical radiology technician performed all scans and analyses to ensure consistency. Regional body composition measurements for arms, legs, and head were distinguished from trunk measurements using anatomical landmarks such as the chin, center of the glenohumeral joint, and femoral neck axis, previously described [14]. The head region was excluded from all analyses. The bone mineral content, bone mineral density, fat-free soft tissue mass, and fat mass were measured for the whole-body and individual regions.

To evaluate fat distribution, the following indices were calculated as fat mass parameters: Trunk-to-appendicular fat ratio was calculated as trunk fat mass divided by appendicular fat mass [15]. Appendicular fat mass was calculated as the sum of bilateral arm and leg fat masses. The trunk-to-leg fat ratio was calculated as trunk fat mass divided by bilateral leg fat mass. The arm-to-leg fat ratio was calculated as total arm fat mass divided by total leg fat mass. The trunk-to-arm fat ratio was calculated as trunk fat mass divided by total arm fat mass. Additionally, the fat mass index (FMI), a measure of whole-body adiposity independent of body size, was calculated as total body fat mass (kg) divided by height squared (m^2^) [16].

### Blood pressure (BP) measurements

Systolic BP (SBP) and diastolic BP (DBP) were measured by an experienced physician using an automated device (BP-103i 2, OMRON COLIN, Tokyo, Japan) as described previously [13]. Measurements were performed at least twice, with the participant seated and the left arm supported at heart level after a minimum of 5 min of rest. The average of the two measurements was used for analysis.

### Questionnaire for pubic hair appearance and sedentary behavior

Data on pubic hair appearance and sedentary behavior were obtained from a self-administered questionnaire. For pubic hair appearance, participants were asked to answer the question, “When did pubic hair growth begin?”, and selected one category from the following choices: “3rd grade,” “4th grade,” “5th grade,” and “not yet.” The number of participants who selected “3rd grade” and “4th grade” was low; therefore, these two categories were combined for the analysis as “less than 5th grade”. Participants were also asked to answer the question, “How much time do you spend doing sedentary activities (e.g., playing video games, using a computer or mobile phone, and watching TV) in a day?”, and selected one category from the following choices: “less than 1 h,” “from 1 h to less than 2 h,” “from 2 h to less than 3 h,” “from 3 h to less than 4 h,” “from 4 h to less than 5 h,” and “5 h and more”. The number of participants who selected “from 4 h to less than 5 h” and “5 h and more” was low; therefore, these two categories were combined for the analysis as “4 h and more.”

### Serum parameters analysis

Blood samples were collected during the same session as the body fat mass measurements, between 8:30 AM and 2:30 PM. Samples were immediately stored at −80 ºC until analysis. Serum levels of adipokine, including leptin and adiponectin, were measured using commercially available kits (for leptin, Quantikine ® Human Leptin, R&D systems, Inc., Minneapolis, USA; for adiponectin, Quantikine ® Human Total Adipokine/Acrp 30, R&D Systems, Inc.) as described previously [16, 17]. Additionally, serum omentin-1 levels were measured using a commercially available kit (BioVendor R&D ®, Brno, Czech Republic). The intra- and inter-assay coefficients of variation were 3.5% and 6.5% for adiponectin, 2.0% and 5.2% for leptin, and 1.0% and 5.7% for omentin-1.

### Statistical analysis

An unpaired *t* test or Mann–Whitney *U*-test was used to evaluate sex differences in participant characteristics. The Peason correlation test was applied to assess associations between omentin-1 levels, serum parameters, and body composition metrics. Multiple regression analysis was used to examine the association between serum omentin-1 levels (outcome) and individual predictors, such as fat mass parameters, fat-free soft tissue mass, and BMI. Fat mass parameters included total and regional fat mass, FMI, and fat distribution ratios (trunk-to-appendicular, trunk-to-leg, arm-to-leg, and trunk-to-arm). The association between serum omentin-1 levels and fat mass or fat-free soft tissue mass was adjusted for age, height, current maternal BMI, sedentary behavior, and pubertal status (as determined by pubic hair appearance). Whereas the association between serum omentin-1 levels and BMI, FMI, or fat distribution ratios was adjusted for age, current maternal BMI, sedentary behavior, and pubertal status (as determined by pubic hair appearance). The mean values of parameters according to tertiles of serum omentin-1 levels were calculated using the general linear model, adjusting for potential confounding factors. Furthermore, multiple linear regression analyses were performed to test trends in fat mass parameters, BMI, waist circumference, and omentin-1 tertiles, with adjustments for potential confounding factors. Statistical significance was defined as *p* < 0.05. All data were analyzed using SPSS statistics Desktop for Japan, version 28.0.0.0 (IBM Japan, Ltd., Tokyo, Japan).

## Results

Among the 521 children in the source of the population, 129 were excluded due to incomplete data. The final study population consisted of 392 participants (192 boys and 200 girls), representing 75.2% of the source population, with a mean age of 11.2 ± 0.3 years. Participants characteristics, stratified by sex, are shown in Table 1. The mean whole-body fat mass was 6.98 ± 3.15 kg among girls and 6.36 ± 3.64 kg among boys (*p* = 0.076). Girls had a higher mean leg fat mass (*p* = 0.014) and trunk-to-arm fat ratio but a lower arm-to-leg fat ratio, compared with boys (*p* < 0.001).

**Table 1:** Participant characteristics

Characteristics	All, *N* = 392	Boys, *N* = 192	Girls, *N* = 200	*p* value^*^
Age (years)	11.2 ± 0.3	11.2 ± 0.3	11.2 ± 0.3	0.701
Height (cm)	142.5 ± 6.7	141.6 ± 6.2	143.4 ± 6.9	0.009
Weight (kg)	35.1 ± 7.2	35.0 ± 7.3	35.3 ± 7.1	0.734
Bone mineral content (g)	968 ± 170	950 ± 139	986 ± 195	0.037
Bone mineral density	0.73 ± 0.04	0.73 ± 0.04	0.73 ± 0.05	0.661
Fat-free soft tissue mass (kg)	24.9 ± 4.1	25.0 ± 4.1	24.8 ± 4.1	0.564
Waist circumference (cm)	62.8 ± 7.1	63.2 ± 7.7	62.5 ± 6.4	0.309
Total fat mass (kg)	6.68 ± 3.41	6.36 ± 3.64	6.98 ± 3.15	0.076
Trunk fat mass (kg)	2.37 ± 1.44	2.26 ± 1.56	2.48 ± 1.31	0.130
Appendicular fat mass (kg)	4.31 ± 2.01	4.11 ± 2.13	4.50 ± 1.88	0.054
Arm fat mass (kg)	1.07 ± 0.55	1.06 ± 0.61	1.08 ± 0.48	0.690
Leg fat mass (kg)	3.24 ± 1.49	3.05 ± 1.53	3.42 ± 1.43	0.014
BMI (kg/m^2^)	17.2 ± 2.5	17.3 ± 2.7	17.0 ± 2.3	0.213
FMI (kg/m^2^)	3.62 ± 1.51	3.52 ± 1.67	3.72 ± 1.34	0.203
Trunk to appendicular fat ratio	0.53 ± 0.09	0.53 ± 0.10	0.54 ± 0.09	0.165
Trunk to leg fat ratio	0.71 ± 0.14	0.71 ± 0.15	0.71 ± 0.13	0.806
Trunk-to-arm ratio	2.17 ± 0.38	2.08 ± 0.37	2.26 ± 0.37	< 0.001
Arm-to-leg fat ratio	0.33 ± 0.05	0.34 ± 0.05	0.32 ± 0.05	< 0.001
Omentin-1 (ng/mL)	509 ± 159	517 ± 165	501 ± 153	0.324
Leptin (ng/mL)	3.01 ± 3.07	2.47 ± 3.05	3.53 ± 3.01	0.001
Adiponectin (μg/mL)	7.84 ± 3.67	7.90 ± 3.82	7.79 ± 3.52	0.777
SBP (mmHg)	106.2 ± 10.5	105.6 ± 10.7	106.8 ± 10.2	0.243
DBP (mmHg)	58.9 ± 7.9	58.2 ± 7.44	59.6 ± 8.19	0.080
Total cholesterol (mg/dL)	180.0 ± 26.1	179.9 ± 27.5	180.0 ± 24.6	0.955
HDL-C (mg/dL)	73.8 ± 15.3	76.0 ± 16.2	71.7 ± 14.2	0.005
LDL-C (mg/dL)	95.3 ± 21.0	93.3 ± 21.7	97.1 ± 20.3	0.070
Current maternal BMI mother (kg/m^2^)	20.8 ± 2.6	20.8 ± 2.63	20.8 ± 2.52	0.953
Sedentary behavior (media use), N (%)				0.915
< 1 h/d	87 (22.2)	41 (21.4)	46 (23.0)	
1–2 h/d	184 (46.9)	95 (49.5)	89 (44.5)	
2–3 h/d	80 (20.4)	31 (16.1)	49 (24.5)	
3–4 h/d	30 (7.7)	17 (8.9)	13 (6.5)	
> 4 h/d	11 (2.8)	8 (4.2)	3 (1.5)	
Pubic hair appearance, N (%)				< 0.001
No appearance	316 (80.6)	183 (95.3)	133 (66.5)	
Grade5	64 (16.3)	9 (4.7)	55 (27.5)	
< Grade5	12 (3.1)	0 (0.0)	12 (6.0)	

Values represent mean ± standard deviation, or N (percentage)

*N* number, *BMI* body mass index, *FMI* fat mass index, *SBP* systolic blood pressure, *DBP* diastolic blood pressure, *HDL-C* high-density lipoprotein cholesterol, *LDL-C* low-density lipoprotein cholesterol

^*^Unpaired t-test or the Mann–Whitney U test was performed

The mean serum levels of leptin were significantly higher (*p* = 0.001), whereas high-density lipoprotein cholesterol (HDL-C) levels were significantly lower in girls than in boys (*p* = 0.005). There was no significant difference in mean serum omentin-1 levels between the sexes (girls, 501 ± 153 ng/mL; boys, 517 ± 165 ng/mL, *p* = 0.324).

Table 2 shows the correlation coefficients between mean serum omentin-1 levels and various covariates in girls and boys. Serum omentin-1 levels demonstrated a significant inverse association with body weight, waist circumference, fat-free soft tissue mass, and fat mass parameters in both sexes, and serum leptin levels in girls. In contrast, omentin-1 levels exhibited a significant positive correlation with serum HDL-C and adiponectin levels in both sexes.

**Table 2:** Correlation coefficients between omenti-1 levels and covariates

	All, *N* = 392		Boys, *N* = 192		Girls, *N* = 200	
	*r*	*p* value	*r*	*p* value	*r*	*p* value
Body weight	−0.238	< 0.001	−0.229	0.001	−0.247	< 0.001
Body height	−0.146	0.004	−0.112	0.120	−0.168	0.017
Bone mineral content	−0.172	< 0.001	−0.190	0.008	−0.158	0.026
Fat-free soft tissue mass	−0.213	< 0.001	−0.204	0.005	−0.227	0.001
Waist circumference	−0.263	< 0.001	−0.242	0.001	−0.298	< 0.001
Total fat mass	−0.223	< 0.001	−0.214	0.003	−0.227	0.001
Trunk fat mass	−0.221	< 0.001	−0.206	0.004	−0.234	0.001
Arm fat mass	−0.246	< 0.001	−0.237	0.001	−0.258	< 0.001
Leg fat mass	−0.207	< 0.001	−0.205	0.004	−0.199	0.005
Appendicular fat mass	−0.220	< 0.001	−0.216	0.003	−0.217	0.002
BMI	−0.234	< 0.001	−0.233	0.001	−0.245	< 0.001
FMI	−0.210	< 0.001	−0.205	0.004	−0.211	0.003
Trunk-to-appendicular fat ratio	−0.126	0.012	−0.095	0.191	−0.156	0.028
Trunk-to-leg fat ratio	−0.148	0.003	−0.120	0.096	−0.180	0.011
Trunk-to-arm fat ratio	−0.007	0.892	0.032	0.661	−0.022	0.754
Arm-to-leg fat ratio	−0.158	0.002	−0.170	0.019	−0.183	0.009
SBP	−0.024	0.636	−0.013	0.861	−0.030	0.670
DBP	−0.001	0.977	−0.032	0.663	0.036	0.618
Total cholesterol	0.140	0.005	0.162	0.025	0.116	0.103
HDL-C	0.240	< 0.001	0.261	< 0.001	0.206	0.003
LDL-C	0.069	0.173	0.076	0.295	0.072	0.313
Adiponectin	0.255	< 0.001	0.200	0.005	0.317	< 0.001
Leptin	−0.192	< 0.001	−0.136	0.060	−0.238	0.001

*p* values were determined using the Person’s correlation test

*N* number, *r* Person’s correlation coefficient, *BMI* body mass index, *FMI* fat mass index, *SBP* systolic blood pressure, *DBP* diastolic blood pressure, *HDL-C* high-density lipoprotein cholesterol, *LDL-C* low-density lipoprotein cholesterol

Table 3 shows the results of the multiple regression analysis examining the associations between the mean serum omentin-1 levels (as the dependent variable) and various body fat parameters (whole-body, trunk, appendicular, arm, leg, fat ratios of trunk-to-appendicular, trunk-to-leg, trunk-to-arm, arm-to-leg, and FMI), or fat-free soft tissue mass and BMI (as independent variables), after adjusting for potential confounding factors. Whole-body and all regional-body fat masses showed significant associations with serum omentin-1 levels across both sexes. Moreover, the trunk-to-appendicular and trunk-to-leg fat ratios in girls showed significant inverse associations with omentin-1 levels. Fat-free soft tissue mass and BMI also showed significant associations with omentin-1 levels.

**Table 3:** Associations of serum omentin-1 levels with fat mass parameters, fat-free soft tissue mass, or BMI

	All, *N* = 392			Boy, *N* = 192			Girl, *N* = 200		
	β	*p* values	VIF	β	*p* values	VIF	β	*p* values	VIF
Total fat mass^a^	−0.208	< 0.001	1.413	−0.185	0.026	1.370	−0.238	0.005	1.451
Trunk fat mass^a^	−0.202	< 0.001	1.362	−0.170	0.038	0.755	−0.241	0.004	1.404
Appendicular fat mass^a^	−0.204	< 0.001	1.428	−0.191	0.023	1.390	−0.226	0.008	1.457
Arm fat mass^a^	−0.240	< 0.001	1.332	−0.211	0.010	1.323	−0.283	< 0.001	1.364
Leg fat mass^a^	−0.184	0.002	1.451	−0.179	0.034	0.711	−0.199	0.019	1.474
FMI^b^	−0.210	< 0.001	1.163	−0.167	0.029	1.163	−0.253	< 0.001	1.162
Trunk to appendicular fat ratio^b^	−0.105	0.043	1.064	−0.047	0.529	1.076	−0.150	0.041	1.068
Trunk to leg fat ratio^b^	−0.133	0.010	1.049	−0.076	0.304	1.070	−0.178	0.014	1.052
Trunk to arm fat ratio^b^	0.039	0.463	1.124	0.084	0.256	1.066	0.010	0.896	1.134
Arm to leg fat ratio^b^	−0.184	< 0.001	1.042	−0.160	0.025	1.004	−0.213	0.003	1.048
Fat-free soft tissue mass^a^	−0.289	0.002	3.343	−0.283	0.034	3.533	−0.329	0.018	3.923
BMI^b^	−0.231	< 0.001	1.146	−0.176	0.023	1.184	−0.281	< 0.001	1.163

*N* number, *β* standardized coefficient, *VIF* variance inflation factor, *FMI* fat mass index, *BMI* body mass index

^a^Multiple regression analysis was used to examine the associations between serum omentin-1 level (outcome) and individual body composition variables (whole-body or regional-body fat, including trunk, appendicular, arms, legs, and fat-free soft tissue mass) (as individual predictors), adjusted for age, height, current maternal body mass index (BMI), sedentary behavior, and pubic hair appearance

^b^Multiple regression analysis was used to examine the associations between serum omentin-1 level (outcome) and individual fat distribution ratios (the trunk-to-appendicular, trunk-to-leg, trunk-to-arm, and arm-to-leg ratios), FMI, or BMI (as individual predictors), adjusted for age, current maternal BMI, sedentary behavior, and pubic hair appearance

**Fig. 1:**
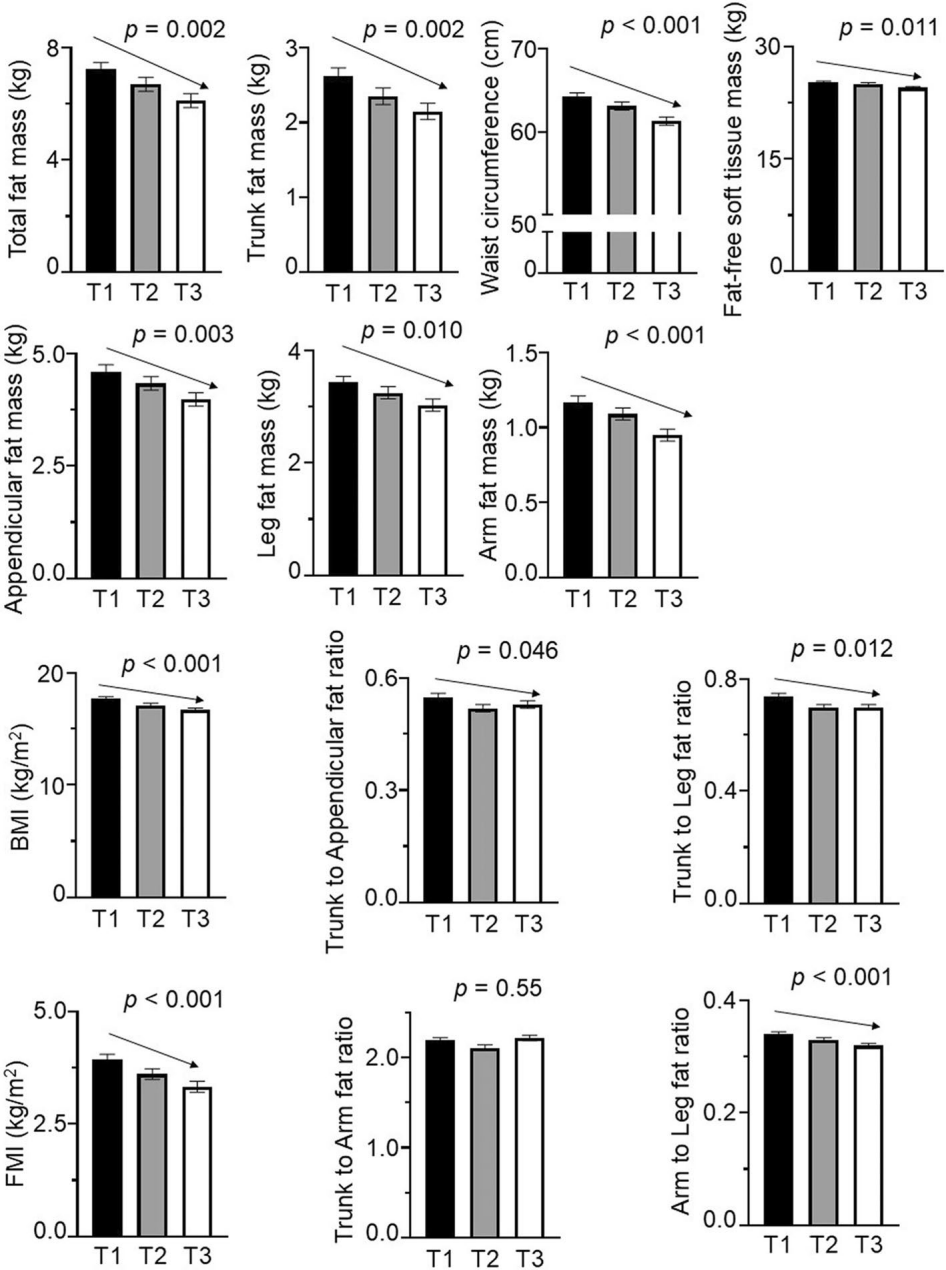
Adjusted means of fat mass parameters, fat-free soft tissue mass, waist circumference, and body mass index (BMI) across tertiles of serum omentin-1 levels. Omentin-1 tertiles were defined as: T1 (181–430 ng/mL), T2 (432–546 ng/mL), and T3 (549–1274 ng/mL). Mean values were calculated after adjusting for potential confounding factors, including age, height, current maternal BMI, sedentary behavior, and pubic hair appearance, using the general liner model. *p*-values were analyzed by trend tests after adjusting for the same potential confounding factors. Arrows indicate statistically significant trends (*p* < 0.05). FMI, fat mass index

Figure 1 presents the adjusted means of body fat parameters, fat-free soft tissue mass, waist circumference, and BMI across tertiles of the mean serum omentin-1 levels. Whole-body fat mass and regional-body fat mass (trunk, appendicular, arms, and legs) significantly decreased from the lowest to the highest tertiles of serum omentin-1 levels. Regional fat ratios, especially the trunk-peripheral fat ratios (trunk-to-appendicular and trunk-to-leg), exhibited modest but significant decreases across tertiles. Similarly, fat-free soft tissue mass, waist circumference, FMI, and BMI significantly decreased as serum omentin-1 levels increased.

## Discussion

To the best of our knowledge, the present study is the first population-based investigation of school children to examine the association between serum omentin-1 levels and various body composition parameters measured using DXA. Our finding revealed significant inverse associations between serum omentin-1 levels and multiple body composition variables, including regional fat mass, FMI, fat-free soft tissue mass, and BMI. School children who had a large amount of fat mass tended to have a lower concentration of serum omentin-1. Moreover, in girls, the trunk-to-peripheral fat ratios, such as the trunk-to-appendicular fat and trunk-to-leg fat ratios, were inversely associated with omentin-1 levels. When compared between girls and boys, girls had significantly higher leg fat mass and a higher ratio of pubic hair appearance. It has also been shown that sexual dimorphism in fat patterning is apparent with girls having considerably more peripheral fat than boys in white Western children [18]. Therefore, these findings in girls may relate to female pubertal development, and the inverse association between omentin-1 levels and trunk-to-peripheral fat ratios in girls may reflect increased peripheral fat deposition. Our results indicate that circulating omentin-1 levels are linked not only to trunk fat mass, particularly VAT but also to other body components such as fat-free soft tissue mass and SAT. However, trunk fat mass may serve as a confounding factor in observed associations between omentin-1 levels and the other body composition components.

BMI is widely used to assess adiposity across all age groups. However, BMI encompasses both fat mass and fat-free mass, leading to limitations in its sensitivity (66%) and high specificity (94%) as a measurement of fatness, which can result in misclassification [19]. To address some of these limitations, the BMI standard deviation score has been proposed as alternative indices for children and adolescents [20]. Despite this, challenges remain in accurately reflecting fat mass. Distinguishing between fat mass and fat-free mass is particularly critical in children, as weight gain during growth is predominantly attributed to fat-free mass rather than fat mass.

DXA scans, as employed in the present study, provide precise regional body composition measurements (e.g., arm, leg, and trunk) with minimal radiation exposure, making them well-suited for assessing adiposity. DXA-measured regional fat distribution, such as the trunk-to-peripheral fat ratio, offers a more accurate evaluation of fat distribution compared to the BMI standard deviation score [20]. Importantly, DXA-measured trunk fat encompasses both VAT and SAT, whereas arm and leg fat does not include VAT. Consequently, parameters such as the trunk-to-appendicular and trunk-to-leg fat ratios serve as weight-independent indicators of the relative amount of VAT. DXA-derived fat ratios offer greater precision in measuring fat distribution compared with conventional anthropometric indices, such as the waist-to-hip ratio.

Epidemiological studies have consistently demonstrated that increased VAT is more strongly associated with obesity-related comorbidities compared to SAT [21, 22]. VAT functions as an endocrine organ, secreting bioactive adipokines, including leptin, adiponectin, and omentin-1 [23]. Previous studies, including our own, have shown a strong positive correlation between serum leptin levels and fat mass in children, whereas adiponectin levels are inversely correlated with fat mass [16, 17, 24, 25]. In our simple correlation analysis, omentin-1 levels exhibited a significant moderate positive correlation with adiponectin levels in girls and a weak positive correlation in boys. These findings align with those of animal studies that reported a positive correlation between omentin-1 and adiponectin levels in diet-induced obesity models [4]. In contrast, the present study observed a significant inverse association between omentin-1 and leptin levels. Notably, a previous small human intervention study demonstrated that pharmacological doses of recombinant leptin administrated to healthy men resulted in reduced omentin-1 levels [26]. Our results are consistent with those of previous studies, suggesting that omentin-1 may have an association with obesity-related comorbidities, similar to other adipokines.

Many studies have reported that mature omentin-1 is released primarily from the VAT [3], whereas SAT does not secrete omentin-1 [2]. However, in contrast to previous studies and their findings, the present study demonstrated inverse associations between omentin-1 levels and both appendicular fat (primarily SAT) and trunk fat (including VAT) in simple correlation analyses. Yang et al. found that omentin-1 mRNA is expressed in stromal vascular cells of omental adipose tissue but not in adipocytes [2]. Experimental evidence suggests that VAT expansion increases the distance between adipocytes and blood vessels, leading to hypoxia and potentially altering the secretion of adipose-derived factors, including omentin-1 and adiponectin [27]. Our findings also show a strong correlation between appendicular fat mass and trunk fat mass in both sexes (correlation coefficient, *r* = 0.95) [16]. This indicates that children with a high peripheral fat mass (predominantly SAT) tend to also have a high trunk fat mass, which includes both VAT and SAT. Therefore, although SAT does not secrete omentin-1 [2], the observed correlation between serum omentin-1 levels and appendicular fat mass may reflect the influence of trunk fat mass as a confounding factor. In multiple regression analyses, the trunk-to-peripheral fat ratios were significantly associated with serum omentin-1 levels, suggesting that trunk fat exerts a greater influence on omentin-1 levels than dose appendicular fat. The trunk-to-extremity fat ratio has been identified as a superior surrogate maker for VAT and SAT [28], highlighting its utility in evaluating the relative contributions of VAT and SAT.

The present study has a few limitations. First, owing to the cross-sectional study design, it could not establish causal or temporal relationships between circulating omentin-1 levels and body fat accumulation. Second, the study lacked data on Tanner scale scores, physical activity, and nutrient intake, which are potential confounding factors influencing the association between serum omentin-1 levels and body compositions in children. Third, the data were obtained from 75.2% of eligible children attending two elementary schools in Hamamatsu, Japan. However, anthropometric measures of the study participants in this study were comparable with those reported in a Japanese national survey data of children aged 11.2 years [29], suggesting that our study population was considered to have similar characteristics to general Japanese children aged 11.2 years.

## Conclusions

Our cross-sectional analysis from this population-based study revealed inverse correlations between serum omentin-1 levels and various body compositions, including whole-body and regional-body fat parameters and lean mass, in Japanese school children. These results suggest that circulating omentin-1 levels are associated not only with trunk fat mass, particularly VAT, but also with other body components, including lean mass and SAT. However, the associations between omentin-1 levels and these body components may be confounded by trunk fat mass. Further studies are needed to clarify the underlying physiological mechanisms regarding omentin-1 and its association with body composition.

## Data Availability

No datasets were generated or analysed during the current study.

## Abbreviations

DXA: Dual-energy X-ray absorptiometry
VAT: Visceral adipose tissue
SAT: Subcutaneous adipose tissue
T2DM: Type 2 diabetes mellitus
BMI: Body mass index
BIA: Bioelectrical impedance analysis
FMI: Fat mass index
BP: Blood pressure
SBP: Systolic BP
DBP: Diastolic BP
HDL-C: High-density lipoprotein cholesterol
LDL-C: Low-density lipoprotein cholesterol
